# Patterns and implications of extensive heterochrony in carnivoran cranial suture closure

**DOI:** 10.1111/jeb.12127

**Published:** 2014-08-04

**Authors:** A Goswami, L Foley, V Weisbecker

**Affiliations:** * Department of Genetics, Evolution & Environment, University College LondonLondon, UK; † Department of Earth Sciences, University College LondonLondon, UK; ‡ School of Biological Sciences, University of QueenslandSt. Lucia, Australia

**Keywords:** Carnivora, ecology, Heterochrony, morphological diversity, phylogeny, sutures

## Abstract

Heterochronic changes in the rate or timing of development underpin many evolutionary transformations. In particular, the onset and rate of bone development have been the focus of many studies across large clades. In contrast, the termination of bone growth, as estimated by suture closure, has been studied far less frequently, although a few recent studies have shown this to represent a variable, although poorly understood, aspect of developmental evolution. Here, we examine suture closure patterns across 25 species of carnivoran mammals, ranging from social-insectivores to hypercarnivores, to assess variation in suture closure across taxa, identify heterochronic shifts in a phylogenetic framework and elucidate the relationship between suture closure timing and ecology. Our results show that heterochronic shifts in suture closure are widespread across Carnivora, with several shifts identified for most major clades. Carnivorans differ from patterns identified for other mammalian clades in showing high variability of palatal suture closure, no correlation between size and level of suture closure, and little phylogenetic signal outside of musteloids. Results further suggest a strong influence of feeding ecology on suture closure pattern. Most of the species with high numbers of heterochronic shifts, such as the walrus and the aardwolf, feed on invertebrates, and these taxa also showed high frequency of closure of the mandibular symphysis, a state that is relatively rare among mammals. Overall, caniforms displayed more heterochronic shifts than feliforms, suggesting that evolutionary changes in suture closure may reflect the lower diversity of cranial morphology in feliforms.

## Introduction

The study of development, and the effects on adult morphology of altering developmental patterns, is fundamental to all aspects of biology and palaeobiology. Changes in the timing and rate of growth during development, termed heterochrony, can generate new morphologies and lead to vast differences in morphological diversity and disparity (Gould, 1977, 1982; Alberch *et al*., 1979; Alberch, 1985; Gilbert *et al*., 1996; Klingenberg, 1998; Nunn & Smith, 1998; Smith, 2001; Sánchez-Villagra *et al*., 2002, 2008; Sears, 2004; Cobb & O'Higgins, 2007; Sears *et al*., 2007; Weisbecker *et al*., 2008; Goswami *et al*., 2009, 2012). Due to the difficulty in obtaining complete developmental series from wild animals, the current understanding of how heterochony may impact morphological evolution comes largely from studies of domesticated or model organisms. The familiar placental order Carnivora in particular has been the focus of many evolutionary studies, with domesticated dogs and cats providing important insights into the relationships among development, selection and diversity (Olsen, 1985; Wayne, 1986; Morey, 1994; Fondon & Garner, 2004; Sears *et al*., 2007; Drake & Klingenberg, 2010).

Skeletal development and the evolutionary consequences of changes in skeletal development are particularly well studied in mammals, because skeletal morphology is a good proxy of whole-animal morphology, and the robustness of bone (e.g. in terms of storage in museum collections) has made skeletal developmental processes the most accessible of all developmental traits available for study. Various approaches to studying mammalian skeletal development are possible, each requiring different sets of data and coming with different complications for comparative analyses. However, the shared aim of many analyses is the identification of heterochrony (changes in developmental timing). Thus, studies of evolutionary changes in timing of bone development may focus on the beginning or end of development, as well as on the rate and allometry of bone development.

In addition to the well established and classically Gouldian analyses of growth heterochrony (Gould, 1982), a very successful approach has emerged that focuses on changes in the sequence of developmental events (Smith, 1997, 2002; Velhagen, 1997; Bininda-Emonds *et al*., 2002; Jeffery *et al*., 2002a,b2002b, 2005). The advantage of sequence-based analyses is that they do not require ageing, staging or sizing data, which often prevent analysis of specimens sourced, for example, from museum collections. Although sequence heterochrony is not always easily classified into the classical categories of paedomorphosis (‘underdevelopment’) and peramorphosis (‘overdevelopment’) and does not quantify rate changes, shifts in developmental sequence can represent fundamental changes in the developmental patterning of an organism or clade. Thus, research into sequence heterochrony can provide insights into an otherwise undetectable type of developmental change whose evolutionary impact stands to be at least as important as that of heterochrony rate changes (Bininda-Emonds *et al*., 2002).

Studies of the beginning of skeletal ossification in mammals are a good example of how sequence heterochrony can inform our understanding of developmental structure changes in large vertebrate radiations. Mammalian ossification sequence heterochrony in particular has been successfully applied to the marsupial–placental dichotomy (Smith, 1997, 2001; Sánchez-Villagra, 2002; Goswami, 2007; Sánchez-Villagra *et al*., 2008; Weisbecker *et al*., 2008; Goswami *et al*., 2009, 2012), developmental synapomorphies of placental superorders (Hautier *et al*., 2010, 2011) and targeted studies of other mammalian clades (Prochel, 2006; Goswami & Prochel, 2007; Prochel *et al*., 2008; Koyabu *et al*., 2011). Ample differences in cranial and post-cranial ossification sequence heterochrony exist between higher clades of mammals, many of which are relatable to specific morphological differences such as unusual limb morphology or even developmental constraints (Sears, 2004; Sánchez-Villagra *et al*., 2008; Weisbecker *et al*., 2008; Goswami *et al*., 2009; Weisbecker, 2011). Recently developed methods have polarized sequence shifts on phylogenetic branches, identifying, for example, that marsupials delay the ossification of their hind limbs (Jeffery *et al*., 2005; Weisbecker *et al*., 2008). For carnivorans, data on the onset of skeletal ossification come mainly from the domestic cat, *Felis catus*, and has been compared to a broad range of other mammals (Sánchez-Villagra *et al*., 2008). Surprisingly, no active heterochronic shifts were identified between the cat and pangolins (scaly anteater), the extant sister group of Carnivora (Springer *et al*., 2005), although more data from other carnivorans are needed to determine if this conservation of timing of onset of ossification characterizes all carnivorans.

Of the three principal aspects of skeletal developmental timing (onset, termination and rate), the timing of the end, or cessation, of bone development remains virtually unknown. Bone may form or remodel well into maturity, as has been noted in the crania of male lions (Benoit, 2010), and thus identifying an endpoint is not straightforward. For the study of long bones, the fusion of epiphyses is a marker of the end of bone development. Similarly, suture closure in cranial bones can be used as a proxy for the end of cranial bone development, although this is further complicated by the observation that some sutures never close completely (Jaslow, 1990). Sutures serve many purposes through development and life, from allowing brain expansion during early growth (Herring & Teng, 2000) to accommodating strain throughout life (Herring, 1972; Wroe, 2008, 2010). Sutures are able to reduce strain locally within the brain case at the expense of elevated strain in other regions (Moazen *et al*., 2009; Jasinoski *et al*., 2010), and strain is found to be elevated in areas where completely closed sutures are located in many vertebrates, although the magnitude and significance of this effect may vary across mammalian taxa (Wang *et al*., 2010). Premature closure of sutures is also found to increase abnormal loading and lead to deformities and irregular bone growth (Persson, 1995). Given the diversity of mammalian skull shapes and sizes, the purpose of allowing cranial expansion and distributing strain across the skull poses interesting questions as to the impact of phylogenetic and functional influences on the evolution of suture closure in mammals.

Some of the earliest studies of suture closure in mammals were conducted, unsurprisingly, in humans (Todd & Lyon, 1924, 1925), where sutures were examined to determine an age order and to investigate cranial synostosis. Cranial synostosis is the result of early fusion of sutures leading to abnormalities of the cranium (Persson, 1995). The brain was thought to be the primary director in the order of suture closure, with vault sutures being the first to close. Krogman (1930) further corroborated this pattern with his study with anthropoid primates in which he identified a general order of suture closure. His sequence of closure is often simplified to: vault, base, circum-meatal, palatal, facial and cranio-facial. The question as to whether this proposed order of closure is as conserved across mammals as suggested by Krogman (1930) has more recently been tested in several mammalian clades, with mixed results.

Studies of hyaenas (Schweiker, 1930), seals (Doutt, 1942), sea lions and fur seals (Brunner *et al.,* 2004), hystricognath rodents (Wilson & Sánchez-Villagra, 2009), chimpanzees and gorillas (Cray *et al*., 2008), anthropoid primates (Chopra, 1957; Flores & Barone, 2012), strepsirrhine primates (Dolan, 1971), suoids (Herring, 1972, 1974), even-toed ungulates (Bärmann & Sánchez-Villagra, 2012) and pteropodid bats (Giannini *et al*., 2006) have shown varied similarity to Krogman's pattern for humans (Krogman, 1930). Some, such as hyaenas, show a relatively similar pattern to humans, whereas others, such as suoids, are quite different. Herring (1972) suggested that differences in these patterns could be attributed to stresses inflicted upon the skull, with peccaries showing early fusion of the palatal and facial regions, which are areas exhibiting high stress due to rooting and feeding behaviours. Sun *et al*. (2004) have also suggested a functional relationship between suture complexity and forces associated with differing life-history traits. Wilson & Sánchez-Villagra (2009) also showed that although the general pattern of suture closure was conserved across hystricognath rodents, there were marked differences between different familial groups, correlated in part with their different life history and locomotory strategies.

To isolate phylogenetic and functional influences on suture closure, it is crucial to examine a clade with dietary and ecological diversity. Carnivora is an ideal group for analysis because it includes species ranging from hypercarnivores to insectivores to folivores, including the aforementioned domestic taxa, and has a well-established phylogeny. Here, we provide new comparative and phylogenetically informed analyses on cranial suture closure across Carnivora to assess (1) if patterns of skull development observed for the domesticated species reflect more general patterns for their broader clades, (2) how allometric and ecological aspects correlate with suture closure timing and patterns and (3) how variable suture closure timing is across Carnivora, and how patterns within carnivorans compare with other mammals. Combined, these analyses will inform on how the cessation of skull development may contribute to cranial morphological diversity in Carnivora and aid in the understanding of the driving forces behind suture closure in mammals as a whole.

## Materials and methods

### Specimens

Degree of suture closure was scored from 390 museum specimens housed at the Natural History Museum (London), the Grant Museum of Zoology (University College London) and the University of Cambridge Museum of Zoology. Twenty-five carnivoran species, representing 13 of the 16 extant family-level clades, were analysed for timing of suture closure (Table 1). For each species, 12–24 specimens were scored, with most species represented by 15 specimens. Specimens ranging from juvenile to adult stages were required for each species to capture the order of suture closure, and species within each family were selected based on the availability of specimens representing multiple developmental stages. For most species, the difference between the youngest and old specimens is at least a doubling in skull length, although a few musteloids were less well sampled (for *A. fulgens, M. putorius* and *P. flavus,* the largest specimens has skull lengths ∼1.5× greater than the smallest specimens). Of the 25 species analysed, the youngest specimens studied for 18 species had no completely closed sutures. Of the seven species in which the youngest specimens had some completely closed sutures (*U. arctos, A. fulgens, L. lutra, M. putorius, P. flavus, O. rosmarus* and *H. ichneumon*), none had more than 10% of sutures closed, and only one had more than 5% of sutures closed. Because gender information was missing from many museum specimens, especially immature specimens, males and females were not separated in the analyses. Captive-bred specimens were excluded, as they may show alterations in cranial bones and sutures as a result of impaired nutrition (Chopra, 1957). Similarly, all specimens with any apparent disorder that could affect results, such as osteoporosis, were also excluded.

**Table 1:** List of species used in analysis, number of specimens per species (N), skull length (in mm) of largest specimen (Slmax), skull length (in mm) of specimen with maximum suture closure (SLspec), maximum suture closure (%) observed in species (MSC), Spearman's rank correlation coefficient between maximum suture closure and skull length (of specimen with maximum suture closure) within each species (Spearman's ρ), Kendall's τ with Krogman's suture closure order (Kendall's τ)

Species	N	SLmax	SLspec	MSC	Spearman's ρ	Kendall's τ
*Nandinia binotata*	15	102	102	93.8	0.86**	0.47**
*Panthera tigris*	24	309	285	54.7	0.62*	0.62**
*Felis silvestris*	15	81.5	81.5	33.6	0.96**	0.40*
*Genetta tigrina*	15	89	87	63.3	0.89**	0.50**
*Civettictis civetta*	15	145	135	76.6	0.93*	0.44*
*Hyaena hyaena*	20	222	206	85.9	0.55*	0.55**
*Proteles cristata*	16	138	120	50.0	0.78**	0.20
*Herpestes ichneumon*	15	103	95	96.9	0.9**	0.46**
*Canis lupus*	12	241	241	61.7	0.3	0.41*
*Vulpes vulpes*	16	167	144	51.6	0.74**	0.36
*Ursus arctos*	16	410	345	60.2	0.59*	0.43*
*Ursus maritimus*	14	373	373	100	0.94**	0.45*
*Melursus ursinus*	17	292	276	93.8	0.41	0.47**
*Ailurus fulgens*	15	102	99	96.9	0.84**	0.39
*Ictonyx striatus*	15	67	60	93.0	0.46	0.35
*Lutra lutra*	15	115.5	115.5	97.7	0.19	0.30
*Mustela putorius*	15	71	71	96.9	0.59*	0.12
*Meles meles*	15	127	125	98.4	0.64**	0.34
*Nasua nasua*	15	121	115	94.5	0.86**	0.40*
*Potos flavus*	15	89	69	98.4	0.39	0.40*
*Procyon lotor*	15	114	105	98.4	0.53*	0.41*
*Odobenus rosmarus*	15	393	378	98.4	0.85**	−0.07
*Callorhinus ursinus*	15	234	219	50.0	0.88**	0.62**
*Mirounga leonina*	15	526	417	14.1	0.41	0.45**
*Halichoerus grypus*	15	298	258	47.7	0.83**	0.55**

* indicates 0.01 < *P* ≤ 0.05.

** indicates *P* ≤ 0.01. Significances for Kendall's τ include a Bonferroni correction for multiple comparisons.

### Suture data collection

For each specimen, a total of 32 sutures were examined for their level of closure (Table 2). Following Wilson & Sánchez-Villagra (2009, Fig. 4), each suture was scored according to the exhibited degree of closure as follows: open (1), ¼ closed (2), ½ closed (3), fully coalesced (4). For a proxy measure of body size, skull length was recorded using digital callipers (0.1 mm resolution), or a measuring tape for large specimens (0.5 mm resolution), as the length from the anterior edge of the interpremaxillary suture to the basion. A subset of skulls from the UCL Grant Museum of Zoology were scored and measured at the beginning and end of the data collection period to confirm that the scoring system remained constant throughout the study.

**Table 2:** List of sutures, their associations in Krogman's (1930) regions, their average closure scores (Avg Closure %) across all carnivoran species analysed, frequency of ‘active’ movements, as determined by a Parsimov analysis (Instances moved), and frequency of ‘passive’ involvement in a shift (when passed by another actively moving event), as determined by a Parsimov analysis (Instances passed)

Suture	Krogman region	Avg Closure (%)	Instances moved	Instances passed
Interfrontal	1 - Vault	38.1	7	24
Fronto-parietal	1 - Vault	40.6	5	31
Interparietal	1 - Vault	52.1	4	13
Supraoccipito-parietal	1 - Vault	47.4	7	24
Basispheno-presphenoid	2 - Cranial Base	26.8	7	28
Basispheno-basioccipital	2 - Cranial Base	46.2	7	22
Exoccipito-basioccipital	2 - Cranial Base	80.5	0	3
Exoccipito-squamosal	2 - Cranial Base	32.3	9	12
Exoccipito-supraoccipital	2 - Cranial Base	81.7	1	1
Alispheno-squamosal	3 - Circum-meatal	42.2	6	22
Alispheno-orbitosphenoid	3 - Circum-meatal	39.8	6	33
Orbitospheno-frontal	3 - Circum-meatal	36.5	5	20
Fronto-squamosal	3 - Circum-meatal	45.1	4	17
Parieto-squamosal	3 - Circum-meatal	46.6	7	15
Supraoccipito-squamosal	3 - Circum-meatal	42.2	7	15
Interpremaxillary	4 - Palatal	33.7	10	15
Intermaxillary	4 - Palatal	33.7	8	34
Premaxillo-maxillary (ventral)	4 - Palatal	41.7	9	22
Interpalatine	4 - Palatal	31.3	7	20
Maxillo-palatine	4 - Palatal	34.0	2	39
Pterygo-palatine	4 - Palatal	57.2	10	16
*Mandibular symphysis*	*4 - Palatal*	*10.5*	*4*	*2*
Premaxillo-maxillary (facial)	5 - Facial	33.5	9	26
Premaxillo-nasal	5 - Facial	20.5	2	17
Internasal	5 - Facial	20.4	3	12
Maxillo-jugal	5 - Facial	30.1	3	21
Maxillo-lacrimal	5 - Facial	29.6	3	22
Naso-frontal	6 - Cranio-Facial	24.5	4	10
Lacrimo-frontal	6 - Cranio-Facial	24.0	5	26
Jugo-squamosal	6 - Cranio-Facial	17.2	2	8
Palato-alisphenoid	6 - Cranio-Facial	35.9	6	18
Palato-orbitosphenoid	6 - Cranio-Facial	25.7	8	7

### Analyses

#### Suture closure level and rank

To generate rank orders for suture closure, the following procedure was followed. Individual suture closure scores per species were calculated by summing the percentage of specimens in which sutures had a given closure score (score 1–4) by the relative closure of that score (0–100% closed). Specifically, the total closure score for a suture is the sum of the percentage of specimens in which that suture was completely closed (obliterated, score 4) and the percentage with ½ closed sutures (score 3) multiplied by 0.5, and the percentage with ¼ closed sutures (score 2) multiplied by 0.25.

Overall % closure = % (score 4) + 0.5 * % (score 3) + 0.25 * % (score 2)

These raw closure scores for each suture were used to generate a rank order of suture closure (Table S1).

Each specimen was also given a total closure score, generated by calculating the overall % closure scores across all 32 sutures for that specimen (as for individual sutures, but calculated across sutures within a specimen rather than across specimens for one suture). Total closure score for each specimen was then compared against specimen skull length, using the nonparametric Spearman's rank correlation coefficient, to assess the relationship between size and suture closure within species.

**Fig. 1:**
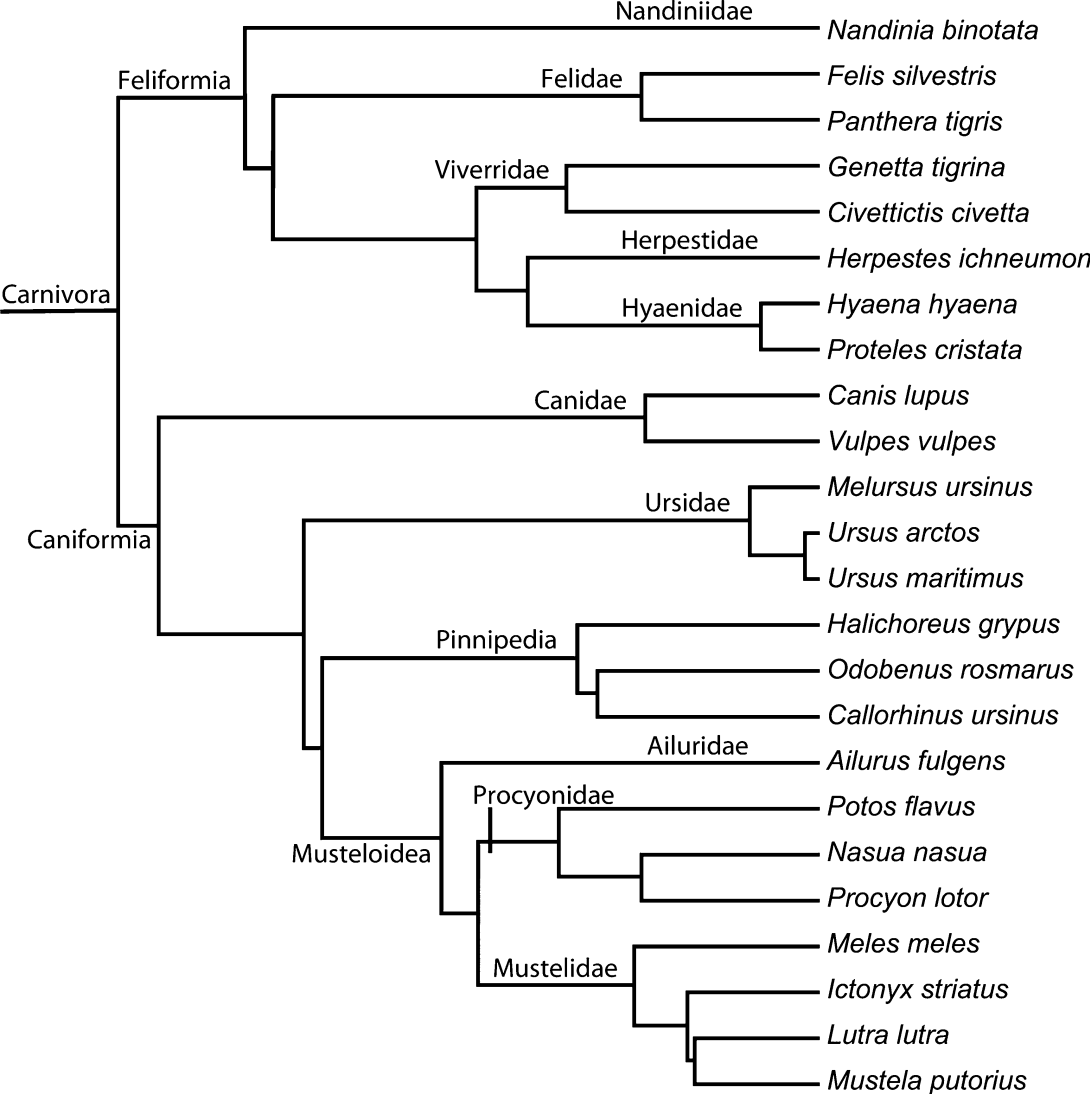
Phylogenetic tree of sampled species used in pgls and Parsimov analyses, based on recent supertree analysis of Carnivora (Nyakatura & Bininda-Emonds, 2012).

To assess the relationship between total suture closure and size across Carnivora, and specifically to test whether suture closure decreases with size as it does in hystricognath rodents and artiodactyls (Wilson & Sánchez-Villagra, 2009; Bärmann & Sánchez-Villagra, 2012), total suture closure and skull length were compared for the specimens showing the maximum suture closure for each species. The relationship between suture closure and skull length was analysed using Spearman's rank correlation coefficient, as well as phylogenetic generalized least squares (pgls, using the ape package in R (Paradis *et al*., 2004)) to assess the relationship independent of the effects of phylogenetic nonindependence. The phylogeny and divergence times for the pgls analysis were based on a recent supertree of Carnivora (Nyakatura & Bininda-Emonds, 2012)(Fig. 1). Brownian motion was assumed as the dominant evolutionary process. The elephant seal, *Mirounga leonina,* was excluded in the pgls analysis, as this species represents an extreme and high-leverage outlier due to the large size of the skull compared with the very low degree of closure.

The order of suture closure was compared pairwise across all carnivoran species using Kendall's tau (τ). Kendall's τ was also used to compare the order of suture closure within Carnivora with the order proposed by Krogman (1930). To generate a comparable ranking for Krogman's order, each of the skull regions was assigned a number corresponding to its relative timing of suture closure as follows: (1) vault sutures, (2) cranial base sutures, (3) circum-meatal sutures, (4) palatal sutures, (5) facial sutures and (6) cranio-facial sutures (Table 2).

Spearman's rank correlation coefficient and Kendall's τ were calculated in PAST (Hammer *et al*., 2001), and a *P* < 0.05 significance value was used in all analyses. Where applicable, a Bonferroni correction was used to adjust for multiple comparisons.

#### Sequence heterochrony

For a more detailed understanding of where heterochronic shifts in suture closure have occurred in carnivoran evolution, event-pair analysis was employed. This involves the comparison of suture closure ranks between all elements, determining for each suture whether it closes before (0), at the same time (1) or after (2) every other suture considered (Smith, 1997; Nunn & Smith, 1998). Sutures that remained open were scored as missing data (‘?’) with respect to each other. Again, *Mirounga leonina* was excluded from analysis because the low degree of suture closure in adults leads to missing data scoring in 46% of event pairs. Krogman's (1930) hypothesized general mammalian sequence of suture closures was used as an outgroup. Event-pair analysis was conducted using the Parsimov algorithm (Jeffery *et al*., 2005). Parsimov determines the most parsimonious occurrence of heterochrony on a phylogeny. This is determined using a consensus of analyses derived from two apomorphy lists of event pairs in PAUP* (based on accelerated transformation [ACCTRAN] and delayed transformation [DELTRAN] character transformation scenarios). The same phylogeny as for pgls was used to obtain the apomorphy lists. This use of the consensus of both analyses results in a robust, but probably conservative, estimate of heterochronic shifts and their polarities (Harrison & Larsson, 2008; Weisbecker *et al*., 2008). Parsimov-based genetic inference, or Pgi, is another, more intricate algorithm for analysis of sequence evolution in a phylogenetic framework (Harrison & Larsson, 2008) that can be less conservative than Parsimov (although results of both analyses are generally quite similar; (Maxwell & Larsson, 2009; Maxwell *et al*., 2010). Our use of the more conservative Parsimov method in this study may result in exclusion of some heterochronic shifts from consideration, but this also ensures that only strongly supported heterochronic changes were recovered.

## Results

### Suture closure scores

**Fig. 2:**
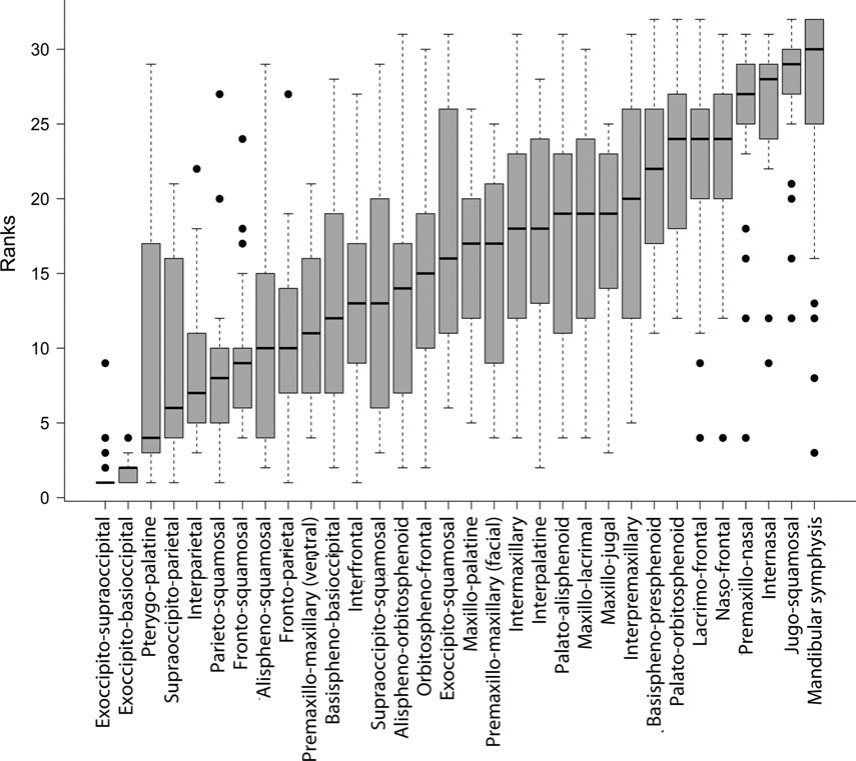
Boxplot of distribution of suture closure ranks across species for each suture investigated, sorted from earliest (low rank number) to latest (high rank number) median suture closure date.

Across the 32 sutures, the mandibular symphysis exhibited the lowest average closure score (10.5%), followed by the jugo-squamosal, internasal, premaxillo-nasal, naso-frontal and lacrimo-frontal sutures (17–24%) (Table 2, Fig. 2). The exoccipito- supraoccital and exocippito-basiocciptal sutures displayed by far the greatest average percentage closure (80–82%). The average closure across all 32 sutures was relatively low, 38%, reflecting the observation that most cranial sutures never close entirely. Average suture rank order and rank variability across all species are shown in Fig. 2.

The specimens showing maximum suture closure were used to generate a suture closure score for each species (Table 1). *Mirounga leonina*, the Northern elephant seal, displayed the lowest maximum suture closure (14%), whereas the musteloids showed the highest maximum suture closures (93–98%). Overall, the feliforms exhibited slightly lower values of maximum closure (mean = 69.5%) when compared with caniforms (mean = 79.5%). However, there is a broad range observed across caniforms, with all musteloids exhibiting total closure of over 90% of sutures, whereas most pinnipeds never close more than half of their cranial sutures.

### Suture closure and skull length

**Fig. 3:**
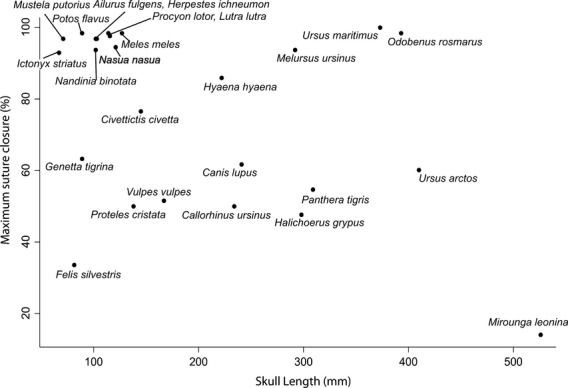
Maximum suture closure (%) against skull length of specimen displaying maximum suture closure (in mm) across Carnivora.

Only six caniforms (*C. lupus, M. ursinus, I. striatus, L. lutra, P. flavus and M. leonina*) of the 25 carnivoran species examined showed no significant correlation between skull length and total suture closure score within species (Table 1). All correlations, including nonsignificant ones, were positive; this consistent result confirms that the cranium becomes increasingly fused with increased size and supports the use of cranial fusion as a common and practical proxy of specimen age for ontogenetic series. Across the 24 species (*M. leonina* being excluded as an outlier), maximum suture closure score showed a weak and nonsignificant tendency to negatively correlate with maximum skull length (Spearman's ρ = −0.247, *P* = 0.234)(Fig. 3), which disappeared after phylogenetic correction (pgls coefficient 1.89, *P* = 0.91).

### Suture closure pattern

Kendall's tau (τ) was used to conduct pairwise comparisons of the order of suture closure across the 25 species studied. Most taxa showed significant correlations in suture closure order prior to a Bonferroni correction, but the correlation ranged widely, from −0.085 to 0.737 (Table S2). Of the 300 comparisons, 76 were still significant after a Bonferroni correction. Many significant correlations were observed between musteloids, but nearly half of the significant correlations were between feliforms and caniforms, suggesting that a strong phylogenetic signal is not present. Only three species (*O. rosmarus*, *P. cristata* and *P. flavus*) showed no significant correlations with other carnivorans.

When patterns of suture closure for each species were compared with the suture closure order for mammals proposed by Krogman (1930), correlations ranged from −0.07 to 0.62 (Table 1). A significant positive correlation with Krogman's order was observed for 22 of the 25 species prior to a Bonferroni correction, and 17 of 25 species after correction. The three taxa that failed to show any significant correlations with other carnivoran species (*O. rosmarus*, *P. cristata* and *P. flavus*) also did not show a significant correlation with Krogman's order.

Most palatal sutures (intermaxillary, interpremaxillary and maxillo-palatine) have relatively average percentage closures in all species (around 34%, compared with an average of 38% across all sutures). Moreover, the closure of the intermaxillary, interpremaxillary and maxillo-palatine were found to be highly variable across Carnivora (Table S1). The highest degree of closure for palatal sutures was exhibited by the pterygo-palatine suture (57%) in *O. rosmarus*. For all of the other pinnipeds in this analysis, including *C. ursinus, M. leonina* and *Halichoerus grypus*, most palatal sutures close late or never at all.

Closure patterns for Carnivora show relatively little fusion of the internasal and premaxillo-nasal sutures (averaging 20.3% and 20.5% across all Carnivora), the premaxillary division of facial sutures tend to close earlier than other facial sutures in other mammals (Krogman, 1930). The main deviants among carnivorans were musteloids, which display early closure of the premaxillary facial sutures and late closure of the circum-meatal sutures.

Little to no closure of the mandibular symphysis was observed in most species, with the notable exceptions of *Melursus ursinus* (60%), *Potos flavus* (90%), *Proteles cristata* (25%), *Odobenus rosmarus* (43%) and *Meles meles* (25%). Interestingly, most of the social-insectivorous carnivorans display some closure of the mandibular symphysis.

As in Krogman's (1930) proposed pattern of suture closure for anthropoids, basicranial sutures were among the first to close, as well as cranial vault sutures. The latter stages of the pattern were similarly conserved with craniofacial and facial sutures being among the last to fuse, although the two groups fuse in mixed, rather than linear, together. Lacrimal sutures are found to close later than observed by Krogman (1930), and more generally, it appears that the cranio-facial sutures start to fuse before facial sutures in many carnivorans.

### Sequence heterochrony

A Parsimov analysis detected heterochronic change of suture closure in nearly all branches of the carnivoran phylogeny, with exception of the branch leading to Viverridae+(Herpestidae +Hyaenidae), the branch leading to Procyonidae, and the branch leading to Pinnipedia (for a complete list of shifts, see Table S3). Thus, even the most-inclusive carnivoran clades, Feliformia and Caniformia, are distinguished by heterochronic shift in suture closure. No shifts were detected in Carnivora relative to the hypothetical outgroup (represented by Krogman's order), probably due to the low resolution of the outgroup sequence. In Caniformia, the only heterochronic shift detected was that of the interpremaxillary suture being delayed with respect to other palatal group sutures (intermaxillary and maxillo-palatine). In contrast, Feliformia was distinguished by a consistent shift in a larger suite of sutures, including two from the cranial base region (early shift of pterygo-palatine and late shift of the basispheno-presphenoid), earlier closure of the naso-frontal suture relative to the lacrimo-frontal and late closure of parietal sutures (frontal-parietal and supraoccipital-parietal). Most, but not all, of these shifts occur relative to other sutures in the same region, rather than between regions.

At the species level, caniform and feliform species average a similar number of shifts (4.5 vs. 3.9, respectively). However, species with the most heterochrony lie mostly within Caniformia and include the walrus *Odobenus rosmarus* (16 movements), the otter *Lutra lutra* (14 shifts), the raccoon *Procyon lotor* (10 shifts), the grizzly bear *Ursus arctos* (eight shifts) and the northern fur seal *Callorhinus ursinus* (eight shifts). The only feliforms with relatively large numbers of shifts include the unusual social-insectivorous hyaenid, the aardwolf *Proteles cristata* (eight shifts) and the African civet *Civettictis civetta* (six shifts).

Some suture closure events are considerably more often heterochronic compared to others (Table 2). Of the seven most common heterochronic shifts in suture closure (occurring eight or more times each across the phylogeny), six relate to suture closure changes within the palatal region (maxillary, premaxillary or palatal bones), with the notable exception of the suture between the maxillary and palatine, which only displayed two shifts. This high variability of palatal suture closures recovered in the Parsimov analysis is consistent with the direct comparison of rank orders discussed above. The least number of heterochronic shifts is displayed by the two earliest-closing sutures (Exoccipital/Supraoccipital and Exoccipital/Basioccipital). Aside from the palatal region, none of Krogman's regions are distinguished by a particular frequency of heterochrony, with all regions including high-shift and low-shift sutures.

Reconciling the rank variability results (Fig. 2) with the Parsimov results (Table 2) reveals some limitations to assessments of rank variability in the sense that high variability does not necessarily mean high levels of heterochrony. In most cases, particularly high or low numbers in shifts reported by the Parsimov analysis correspond well with high or low rank variability. However, low rank variability of highly heterochronic sutures can occur when the relevant suture does not move relative to many other sutures or due to low resolution in a data set. An example of this is the two premaxillary/maxillary sutures, which shift frequently but mostly relative to 1–3 other sutures and correspondingly show only low rank variation. Conversely, a rarely heterochronic suture can display considerable rank variability if it is often passively involved in a shift (e.g. the alisphenoid/orbitosphenoid suture, which actively shifts only six times but is ‘passed’ by other closure events 33 times). This effect also leads to the superficial impression that the maxillo-palatine suture is highly heterochronic, when in fact it is only involved passively in a very large number of heterochronic shifts (2 active vs. 39 passive shifts).

## Discussion

In contrast to early bone ossification and bone allometry through ontogeny, the cessation of bone growth, here approximated with a measure of suture closure, has received relatively little attention in studies of evolutionary development. As discussed above, sutures serve manifold purposes in allowing growth and accommodating strain through development and life, and thus are of interest for understanding the influences of phylogeny, function and development on anatomy. Here, we analysed timing of cranial suture closure across a suite of morphologically and ecologically diverse carnivorans to assess if suture closure is heterochronic or conservative across taxa, both within Carnivora and compared with hypothesized mammalian suture closure patterns. We also assess the relationship between skull length and suture closure, to test if the positive correlation observed between these variables in other mammalian clades (Wilson & Sánchez-Villagra, 2009; Bärmann & Sánchez-Villagra, 2012) also applies to Carnivora.

As would be expected by any student of mammalian skulls, results show that many sutures never close completely, with dentary, facial and craniofacial sutures showing the least closure across Carnivora. Musteloids show the highest suture closure, with many species closing nearly all cranial sutures. Indeed, it is possible that sutures may close before brain growth is completed in many musteloids, as the morphology of the brain and several markers, such as the lateral sulcus, are clearly visible as a cast on the external surface of the skull; further data are needed to assess the cause of this unusual feature of musteloid crania.

Within most species, suture closure increases with size, reflecting the expectation that sutures close as an animal ages. Surprisingly, this pattern does not extend above the species level, with no significant relationship between level of closure and skull length across carnivorans. This result contrasts with previous analyses in hystricognath rodents (Wilson & Sánchez-Villagra, 2009) and artiodactyls (Bärmann & Sánchez-Villagra, 2012) and suggests that the relationship between suture closure and size is more variable across mammals than previously thought. The best example of this is the complete closure of walrus skulls and extremely low closure of similar-sized elephant seals, or the 100% closure of grizzly bears compared with 50% closure in the closely related and similar-sized polar bears. It is possible that the previously supported inverse relationship between suture closure and skull size is a general mammalian pattern that is obliterated by the diverse ecologies and related cranial adaptations and biomechanical demands on suture closure within carnivores.

Of 300 comparisons of suture rank order between carnivoran species, only 70 showed significant correlations, and, with the exception of many correlations among musteloids, little phylogenetic signal was evident. This result is consistent with the results of the Parsimov analysis, which, despite its conservativeness, detected heterochrony in nearly all branches of the carnivoran phylogenetic tree. At the base of the tree, carnivorans displayed late closure of facial sutures as compared with other mammals, which Parsimov analysis indicated was a passive shift driven by early shifts of craniofacial sutures, rather than active shifts of the facial sutures themselves. Feliformia, Caniformia, and most superfamily- and family-level clades displayed heterochronic shifts, ranging from one shift (Caniformia) to five (Feliformia), and averaging three shifts per clade. Combined with the rank variability plots, this suggests considerable flexibility of suture closure arrangements within Carnivora.

These patterns, and the factors underlying them, are clearly complex, because carnivorans displayed heterochronies in the more-inclusive branches (e.g. the interpremaxillary suture closes after the intermaxillary and the maxillo-palatine sutures in all Caniformia, but before these bones in nearly all Feliformia) as well as between relatively closely related clades and species. As the example above demonstrates, many, but certainly not all, of the heterochronic shifts occur within Krogman's regions (e.g. palatal sutures move earlier or later than other palatal suture) rather than crossing regions. Further analyses are required to test this hypothesis, but these results may suggest that there is significant modularity in cranial suture closure, similar to modularity in the ossification of the mammalian post-cranial skeleton (Goswami *et al*., 2009; Hautier *et al*., 2010), but in contrast to the lack of any evidence of modularity in timing of cranial bone ossification (Goswami, 2007; Goswami *et al*., 2009).

Some suture closure events are far more heterochronic across carnivorans than are others. In contrast to expectations based on other clades (Krogman, 1930), the sutures of the palatal region displayed a disproportionate amount of shifts. Herring (1972) suggested that early closure of palatal sutures may be linked to high stress. Among carnivorans, palatal sutures showed both early and late shifts in timing, without a clear relationship to feeding strategy. Most canids and ursids, as well as the otter, *L. lutra*, showed early shifts in palatal sutures, whereas most felids and hyaenids showed late shifts. Among pinnipeds, palatal sutures showed both early and late shifts, so no particular pattern related to an aquatic lifestyle is evident from the Parsimov analysis. However, the raw suture closure scores for the walrus (*O. rosmarus*) showed that palatal sutures closed more completely in the walrus than in other pinnipeds, which may relate to the development of the domed palate for their specialized method of suction feeding on molluscs.

Another interesting suture is the mandibular symphysis. Although this suture remains open in nearly every species, the four of the five species in which it closes entirely are specialized or predominantly invertivores, including the social-insectivorous species *Melursus ursinus* and *Proteles cristata*, the omnivorous but mostly worm-eating *Meles meles* and the molluscivorous walrus. The increased fusion of the mandibular symphysis in insectivorous carnivorans appears suggest a functional signal; however, it is unusual in light of the high flexibility of the mandibular symphysis in many noncarnivoran insectivorous mammals, such as anteaters and echidnas, which themselves are highly derived in jaw morphology. A fused mandibular symphysis in insectivorous species is very unusual and contradicts the well-supported tenet that low-resistance diets generally favour an open symphysis (Scott *et al*., 2012), although it is worth noting that not all invertivorous diets are necessarily low resistance (e.g. hard-shelled molluscs if not processed with suction feeding). It has also already been established that caniform carnivores in particular do not follow this otherwise generally applicable rule (Scott *et al*., 2012). One intriguing possible explanation for the exceptional symphyseal fusion patterns within Carnivora is that carnivoran jaw biomechanics may differ from that of mammals to such a degree that symphyseal fusion has a different effect in this clade compared with others. Previous study has suggested that variation in sympheseal fusion may be linked to occlusal arrangements during the chewing power stroke in various mammals (Lieberman & Crompton, 2000), although carnivorans were not included in that study. Another factor is that jaws are important in functions other than feeding, such as fighting or prey acquisition (e.g. killing behaviour), and the fusion of the mandibular symphysis may relate to behaviours other than food processing. In addition, even specialized invertivores may occasionally rely on alternative sources of nourishment, such as plants or vertebrates, complicating simple extrapolations of form and function. Further study of jaw biomechanics in carnivorans, including insectivorous and aquatic carnivorans and suture morphology, as well as overall level of fusion (e.g. Byron, 2009), is needed to better understand the functional and evolutionary consequences of symphyseal fusion in this clade.

Beyond the possible functional link between mandibular symphysis closure and an invertivorous diet, our analyses support the existence of a more general interaction between feeding adaptations or lifestyle and heterochronic shifts in suture closure patterns. A simple comparison of the number of heterochronic shifts displayed by taxa show that high numbers of shifts are associated with unusual ecologies. Specifically, of the six taxa showing eight or more heterochronic shifts, three are aquatic carnivorans (*O. rosmarus*, *L. lutra* and *C. ursinus*) and one is the unusual insectivorous hyaenid *P. cristata*.

A comparison between the two largest pinnipeds, the elephant seal *M. leonina* and the walrus *O. rosmarus,* is perhaps the most striking within the data set. *O. rosmarus* has nearly all cranial sutures closed, whereas *M. leonina* displays the lowest degree of suture closure across Carnivora. This difference possibly reflects the walrus’ foraging style of using their snout for digging prey and suction feeding to extract the bivalves that make up most of their diet. By contrast, the southern elephant seal, whose sutures mostly remain open, feeds mostly on cephalopods and other comparatively soft prey. A high degree of suture closure heterochrony and fundamentally differing overall closure (100% vs. 50%) were also evident between the closely related omnivorous Grizzly bear and carnivorous polar bear. However, feeding adaptation is clearly not the only factor in the evolution of suture closure heterochrony, for example, two of the species with the most heterochronies are generalists, the raccoon and the civet cat (10 and 6 shifts respectively), which are not particularly different in terms of size or life history compared with their closest relatives sampled in this study.

Considering the clear correspondence of lifestyle and closure patterns, the Pinnipedia stand out as being one of the few clades without any heterochronic shifts reported to unite the clade. As there is a very high degree of interspecific heterochrony among the pinnipeds sampled, and all pinniped species display high numbers of shift, the lack of any heterochronic shifts that are common across pinnipeds (i.e. resolved at the base of the clade) is probably due to the fast evolution of suture closure patterns masking the underlying ancestral patterns within a clade. Despite not sharing any specific heterochronic shifts, three of the four pinnipeds in this study (all except for *O. rosmarus*) showed the lowest overall suture closure scores across carnivorans. Whether this pattern applies across the rest of Pinnipedia remains to be tested, but it raises the possibility that the transition to an aquatic lifestyle involves paedomorphosis of skeletal development, in this case manifested as low levels of suture closure, in pinnipeds, as has been previously suggested for some cetaceans (Mellor *et al*., 2009; Galatius, 2010; Galatius *et al*., 2011).

Overall, there are more species with extensive heterochrony within Caniformia than in Feliformia. Feliformia also had a greater amount of heterochronies at the base of the clade, suggestive of an overall more conserved feliform pattern. Although there are more caniforms than feliforms represented in this study, the full phylogenetic breadth of these sister clades was sampled. Moreover, sampling alone cannot explain the concentration of heterochronic shifts at the base of Feliformia and other higher level feliform clades, in contrast to more species-specific heterochronic shifts observed among caniforms.

Despite relatively similar times of domestication of cats (Driscoll *et al*., 2007) and dogs (Olsen, 1985; Pang *et al*., 2009), domesticated dog breeds show much greater morphological diversity (Drake & Klingenberg, 2010; Drake, 2011) than domesticated cats, and differences in skull allometry and heterochrony have long been implicated in their disparate evolutionary diversities (Wayne, 1986; Fondon & Garner, 2004; Sears *et al*., 2007). These differences in skull development and diversity between cats and dogs have often been extrapolated to their large subordinal clades, Feliformia (cats, hyaenas, civets, genets, mongooses and Malagasy carnivorans) and Caniformia (dogs, bears, seals, sea lions, walruses, red pandas, racoons, weasels, badgers and otters) respectively (e.g. Sears *et al*., 2007). However, little to no developmental data have been analysed previously for any carnivorans other than cats and dogs and their closest relatives, raising the question of whether patterns in these species can be scaled up to their larger clades.

Our results are consistent with the suggestion that the very different degrees of cranial diversity within domesticated dogs and cats are related to differences in ontogenetic diversity and can be extrapolated to the caniform and feliform radiations. As exemplified by the high morphological diversity of the domestic dog compared with that of the domestic cat, simple heterochronic shifts may have great effects on large-scale patterns of evolution. While we do not have adequate data at present to test the role of onset of ossification, the results presented here suggest that heterochronic shifts at the end of cranial development may facilitate morphological change and contribute to the apparent differences in morphological diversity between the two major branches of Carnivora.

## Supporting information

**Table S1** Suture closure ranks for each species used in analyses. **Table S2** Pairwise rank comparisons across carnivoran species, with Kendall's τ in the lower triangle and significance values in the upper triangle. **Table S3** Complete list of heterochronic shifts in suture closure timing as determined by a Parsimov analysis.
