# Supplementary material for: Patterns and implications of extensive heterochrony in carnivoran cranial suture closure
Source: J Evol Biol. 2014 Aug 4;26(6):1294–306. doi: 10.1111/jeb.12127 (PMC4166780; doi:10.1111/jeb.12127)
Supplement: Supplementary file 1 [file jeb0026-1294-SD1.docx]

Table S1. Suture rank scores for 25 carnivoran species.

|  | *Canis lupus* | *Vulpes vulpes* | *Ursus arctos* | *Ursus maritimus* | *Melursus ursinus* | *Ailurus fulgens* | *Ictonyx striatus* | *Lutra lutra* | *Mustela putorius* | *Meles meles* |
| --- | --- | --- | --- | --- | --- | --- | --- | --- | --- | --- |
| Mandibular symphysis | 29 | 30 | 32 | 31 | 13 | 32 | 32 | 32 | 32 | 32 |
| Interpremaxillary | 25 | 26 | 30 | 27 | 31 | 14 | 25 | 29 | 25 | 31 |
| Premaxillo-maxillary (v) | 14 | 11 | 9 | 18 | 8 | 5 | 16 | 19 | 4 | 10 |
| Intermaxillary | 23 | 24 | 17 | 18 | 10 | 5 | 23 | 19 | 4 | 10 |
| Maxillo-palatine (v) | 18 | 14 | 9 | 18 | 18 | 5 | 13 | 26 | 20 | 10 |
| Interpalatine | 28 | 26 | 17 | 18 | 10 | 8 | 14 | 2 | 20 | 6 |
| Pterygo-palate | 17 | 21 | 9 | 18 | 29 | 1 | 1 | 2 | 4 | 10 |
| Palate-alisphenoid | 10 | 7 | 23 | 15 | 20 | 26 | 11 | 11 | 20 | 26 |
| Basispheno-basioccipital | 12 | 3 | 9 | 9 | 16 | 21 | 16 | 19 | 20 | 20 |
| Basispheno-presphenoid | 27 | 17 | 29 | 30 | 28 | 22 | 23 | 11 | 26 | 26 |
| Alispheno-squamosal | 4 | 5 | 28 | 10 | 3 | 12 | 4 | 2 | 4 | 10 |
| Alispheno-orbitosphenoid | 9 | 5 | 17 | 6 | 15 | 16 | 5 | 2 | 4 | 20 |
| Orbitospheno- frontal | 15 | 9 | 16 | 15 | 21 | 18 | 11 | 2 | 4 | 16 |
| Palato-orbitosphenoid | 13 | 16 | 26 | 18 | 21 | 27 | 31 | 24 | 30 | 28 |
| Maxillo-lacrimal (o) | 19 | 14 | 7 | 4 | 23 | 12 | 16 | 11 | 4 | 20 |
| Jugo-squamosal | 29 | 21 | 31 | 32 | 30 | 30 | 25 | 28 | 20 | 16 |
| Lacrimo-frontal (o) | 21 | 24 | 25 | 26 | 26 | 28 | 21 | 11 | 4 | 20 |
| Maxillo-jugal | 22 | 20 | 23 | 15 | 24 | 23 | 14 | 24 | 3 | 6 |
| Premaxillo-maxillary (f) | 20 | 21 | 6 | 10 | 9 | 18 | 16 | 19 | 4 | 6 |
| Premaxillo-nasal | 29 | 29 | 26 | 18 | 31 | 29 | 25 | 30 | 27 | 16 |
| Internasal | 29 | 30 | 22 | 29 | 24 | 24 | 28 | 31 | 29 | 29 |
| Naso-frontal | 23 | 30 | 20 | 18 | 27 | 24 | 22 | 26 | 4 | 20 |
| Interfrontal | 25 | 26 | 9 | 27 | 17 | 15 | 9 | 11 | 1 | 3 |
| Fronto-parietal | 16 | 19 | 9 | 10 | 12 | 8 | 6 | 2 | 1 | 5 |
| Interparietal | 6 | 13 | 4 | 6 | 5 | 11 | 8 | 11 | 4 | 10 |
| Frontal-squamosal | 10 | 17 | 15 | 14 | 5 | 8 | 6 | 10 | 4 | 6 |
| Parieto-squamosal | 8 | 11 | 20 | 10 | 7 | 4 | 9 | 1 | 4 | 4 |
| Supraoccipito-parietal | 5 | 9 | 3 | 3 | 4 | 16 | 16 | 11 | 4 | 16 |
| Supraoccipito-squamosal | 3 | 4 | 5 | 5 | 14 | 20 | 29 | 19 | 28 | 20 |
| Exoccipito- squamosal | 6 | 8 | 7 | 6 | 18 | 31 | 30 | 11 | 31 | 30 |
| Exoccipito- supraoccipital | 1 | 1 | 1 | 1 | 1 | 2 | 1 | 9 | 4 | 1 |
| Exoccipito-basioccipital | 1 | 2 | 2 | 2 | 2 | 3 | 1 | 2 | 4 | 2 |

Table S1 (cont.)

|  | *Nasua nasua* | *Potos flavus* | *Procyon lotor* | *Odobenus rosmarus* | *Callorhinus ursinus* | *Mirounga leonina* | *Halichoreus grypus* | *Nandinia binotata* | *Felis silvestris* |
| --- | --- | --- | --- | --- | --- | --- | --- | --- | --- |
| Mandibular symphysis | 32 | 3 | 32 | 16 | 29 | 12 | 27 | 32 | 25 |
| Interpremaxillary | 27 | 15 | 15 | 18 | 12 | 12 | 9 | 5 | 25 |
| Premaxillo-maxillary (v) | 6 | 5 | 8 | 12 | 16 | 12 | 4 | 4 | 17 |
| Intermaxillary | 14 | 24 | 15 | 18 | 23 | 12 | 18 | 8 | 22 |
| Maxillo-palatine (v) | 11 | 12 | 14 | 21 | 26 | 12 | 24 | 16 | 9 |
| Interpalatine | 11 | 27 | 24 | 22 | 19 | 12 | 27 | 15 | 19 |
| Pterygo-palate | 3 | 21 | 23 | 6 | 11 | 3 | 18 | 3 | 3 |
| Palate-alisphenoid | 17 | 31 | 22 | 25 | 23 | 12 | 27 | 28 | 12 |
| Basispheno-basioccipital | 23 | 28 | 8 | 15 | 2 | 12 | 6 | 24 | 7 |
| Basispheno-presphenoid | 19 | 32 | 18 | 17 | 19 | 12 | 18 | 30 | 14 |
| Alispheno-squamosal | 4 | 15 | 4 | 29 | 10 | 12 | 16 | 9 | 10 |
| Alispheno-orbitosphenoid | 14 | 21 | 4 | 31 | 16 | 12 | 17 | 7 | 14 |
| Orbitospheno- frontal | 19 | 15 | 4 | 30 | 16 | 5 | 14 | 16 | 19 |
| Palato-orbitosphenoid | 26 | 20 | 30 | 32 | 19 | 12 | 22 | 29 | 14 |
| Maxillo-lacrimal (o) | 16 | 24 | 25 | 4 | 27 | 12 | 12 | 22 | 22 |
| Jugo-squamosal | 29 | 30 | 29 | 28 | 29 | 12 | 27 | 31 | 25 |
| Lacrimo-frontal (o) | 19 | 24 | 21 | 9 | 23 | 12 | 13 | 25 | 25 |
| Maxillo-jugal | 23 | 9 | 19 | 14 | 14 | 12 | 25 | 16 | 13 |
| Premaxillo-maxillary (f) | 17 | 4 | 4 | 7 | 22 | 10 | 21 | 25 | 17 |
| Premaxillo-nasal | 29 | 23 | 28 | 4 | 29 | 12 | 27 | 27 | 25 |
| Internasal | 31 | 29 | 31 | 9 | 29 | 12 | 25 | 23 | 25 |
| Naso-frontal | 23 | 15 | 27 | 12 | 27 | 12 | 27 | 20 | 25 |
| Interfrontal | 7 | 9 | 13 | 8 | 15 | 8 | 15 | 10 | 25 |
| Fronto-parietal | 9 | 12 | 8 | 9 | 7 | 4 | 5 | 12 | 19 |
| Interparietal | 7 | 7 | 8 | 18 | 12 | 6 | 10 | 12 | 22 |
| Frontal-squamosal | 4 | 9 | 8 | 24 | 9 | 9 | 6 | 6 | 10 |
| Parieto-squamosal | 10 | 12 | 3 | 27 | 7 | 12 | 8 | 11 | 4 |
| Supraoccipito-parietal | 11 | 6 | 20 | 1 | 4 | 6 | 3 | 21 | 6 |
| Supraoccipito-squamosal | 22 | 8 | 15 | 23 | 5 | 10 | 11 | 14 | 5 |
| Exoccipito- squamosal | 28 | 15 | 26 | 26 | 6 | 12 | 23 | 19 | 7 |
| Exoccipito- supraoccipital | 1 | 2 | 1 | 3 | 1 | 1 | 1 | 1 | 2 |
| Exoccipito-basioccipital | 1 | 1 | 1 | 1 | 2 | 2 | 1 | 1 | 1 |

Table S1 (cont.)

|  | *Panthera tigris* | *Genetta tigrina* | *Civettictis civetta* | *Hyaena hyaena* | *Proteles cristatus* | *Herpestes ichneumon* |
| --- | --- | --- | --- | --- | --- | --- |
| Mandibular symphysis | 27 | 27 | 23 | 31 | 8 | 32 |
| Interpremaxillary | 24 | 8 | 6 | 20 | 9 | 9 |
| Premaxillo-maxillary (v) | 21 | 11 | 11 | 16 | 7 | 19 |
| Intermaxillary | 31 | 20 | 18 | 23 | 12 | 4 |
| Maxillo-palatine (v) | 17 | 24 | 20 | 21 | 18 | 11 |
| Interpalatine | 16 | 20 | 27 | 28 | 13 | 13 |
| Pterygo-palate | 3 | 3 | 3 | 7 | 3 | 3 |
| Palate-alisphenoid | 20 | 19 | 6 | 4 | 5 | 14 |
| Basispheno-basioccipital | 5 | 9 | 11 | 5 | 4 | 15 |
| Basispheno-presphenoid | 22 | 26 | 21 | 17 | 13 | 24 |
| Alispheno-squamosal | 14 | 15 | 4 | 12 | 18 | 20 |
| Alispheno-orbitosphenoid | 10 | 12 | 9 | 15 | 22 | 17 |
| Orbitospheno- frontal | 10 | 6 | 14 | 19 | 29 | 21 |
| Palato-orbitosphenoid | 18 | 13 | 19 | 26 | 26 | 25 |
| Maxillo-lacrimal (o) | 26 | 27 | 26 | 24 | 9 | 30 |
| Jugo-squamosal | 27 | 27 | 31 | 30 | 31 | 31 |
| Lacrimo-frontal (o) | 29 | 27 | 31 | 32 | 30 | 26 |
| Maxillo-jugal | 19 | 23 | 24 | 25 | 13 | 21 |
| Premaxillo-maxillary (f) | 22 | 20 | 24 | 9 | 17 | 23 |
| Premaxillo-nasal | 29 | 27 | 29 | 29 | 27 | 28 |
| Internasal | 31 | 24 | 27 | 26 | 23 | 28 |
| Naso-frontal | 25 | 27 | 30 | 21 | 31 | 26 |
| Interfrontal | 12 | 10 | 14 | 13 | 23 | 17 |
| Fronto-parietal | 14 | 14 | 13 | 18 | 27 | 15 |
| Interparietal | 4 | 3 | 5 | 3 | 6 | 5 |
| Frontal-squamosal | 9 | 6 | 6 | 10 | 18 | 10 |
| Parieto-squamosal | 8 | 5 | 10 | 8 | 9 | 7 |
| Supraoccipito-parietal | 6 | 18 | 17 | 6 | 18 | 8 |
| Supraoccipito-squamosal | 7 | 16 | 21 | 10 | 13 | 6 |
| Exoccipito- squamosal | 13 | 17 | 16 | 13 | 23 | 11 |
| Exoccipito- supraoccipital | 1 | 1 | 1 | 1 | 1 | 1 |
| Exoccipito-basioccipital | 2 | 2 | 2 | 1 | 1 | 1 |

Table S2. Pairwise rank comparisons across carnivoran species, with Kendall’s τ in the lower triangle and significance values in the upper triangle. Values in bold are significant at the p ≤ 0.05 after a Bonferroni correction.

|  | *C. lupus* | *V. vulpes* | *U. arctos* | *U. maritimus* | *M. ursinus* | *A. fulgens* | *I. striatus* | *L. lutra* | *M. putorius* |
| --- | --- | --- | --- | --- | --- | --- | --- | --- | --- |
| *Canis lupus* |  | **0.0000** | 0.0003 | **0.0000** | **0.0000** | 0.0195 | 0.0005 | 0.0003 | 0.1572 |
| *Vulpes vulpes* | 0.74 |  | 0.0021 | **0.0000** | 0.0010 | 0.0962 | 0.0064 | 0.0021 | 0.4775 |
| *Ursus arctos* | 0.45 | 0.38 |  | **0.0000** | 0.0005 | 0.0015 | 0.0110 | 0.0294 | 0.0249 |
| *Ursus maritimus* | 0.72 | 0.61 | 0.62 |  | 0.0002 | 0.0286 | 0.0025 | 0.0012 | 0.0507 |
| *Melursus ursinus* | 0.54 | 0.41 | 0.43 | 0.47 |  | 0.0012 | 0.0022 | 0.0032 | 0.0524 |
| *Ailurus fulgens* | 0.29 | 0.21 | 0.39 | 0.27 | 0.40 |  | **0.0000** | 0.0004 | **0.0006** |
| *Ictonyx striatus* | 0.43 | 0.34 | 0.32 | 0.38 | 0.38 | 0.56 |  | **0.0000** | **0.0000** |
| *Lutra lutra* | 0.45 | 0.38 | 0.27 | 0.40 | 0.37 | 0.44 | 0.61 |  | **0.0006** |
| *Mustela putorius* | 0.18 | 0.09 | 0.28 | 0.24 | 0.24 | 0.43 | 0.60 | 0.42 |  |
| *Meles meles* | 0.19 | 0.11 | 0.35 | 0.26 | 0.39 | 0.55 | 0.58 | 0.39 | 0.61 |
| *Nasua nasua* | 0.43 | 0.30 | 0.43 | 0.34 | 0.53 | 0.70 | 0.72 | 0.60 | 0.53 |
| *Potos flavus* | 0.34 | 0.16 | 0.41 | 0.33 | 0.46 | 0.25 | 0.17 | 0.07 | 0.25 |
| *Procyon lotor* | 0.47 | 0.46 | 0.33 | 0.42 | 0.47 | 0.50 | 0.58 | 0.47 | 0.45 |
| *Odobenus rosmarus* | -0.08 | -0.14 | 0.31 | 0.07 | 0.03 | 0.13 | 0.09 | -0.07 | 0.21 |
| *Callorhinus ursinus* | 0.61 | 0.53 | 0.45 | 0.56 | 0.41 | 0.37 | 0.39 | 0.44 | 0.24 |
| *Mirounga leonina* | 0.35 | 0.23 | 0.56 | 0.37 | 0.35 | 0.40 | 0.47 | 0.41 | 0.39 |
| *Halichoreus grypus* | 0.48 | 0.42 | 0.45 | 0.43 | 0.42 | 0.46 | 0.32 | 0.37 | 0.33 |
| *Nandinia binotata* | 0.33 | 0.22 | 0.36 | 0.27 | 0.38 | 0.64 | 0.53 | 0.38 | 0.38 |
| *Felis silvestris* | 0.64 | 0.61 | 0.40 | 0.52 | 0.39 | 0.34 | 0.37 | 0.39 | 0.06 |
| *Panthera tigris* | 0.60 | 0.54 | 0.48 | 0.54 | 0.42 | 0.35 | 0.52 | 0.48 | 0.22 |
| *Genetta tigrina* | 0.48 | 0.40 | 0.35 | 0.36 | 0.39 | 0.48 | 0.46 | 0.44 | 0.24 |
| *Civettictis civetta* | 0.52 | 0.45 | 0.25 | 0.35 | 0.42 | 0.47 | 0.53 | 0.43 | 0.20 |
| *Hyaena hyaena* | 0.60 | 0.53 | 0.52 | 0.52 | 0.43 | 0.37 | 0.45 | 0.39 | 0.21 |
| *Proteles cristatus* | 0.25 | 0.28 | 0.24 | 0.24 | 0.30 | 0.39 | 0.22 | 0.14 | 0.02 |
| *Herpestes ichneumon* | 0.50 | 0.35 | 0.45 | 0.40 | 0.40 | 0.56 | 0.40 | 0.37 | 0.18 |

Table S2 (cont.)

|  | *M. meles* | *N. nasua* | *P. flavus* | *P. lotor* | *O. rosmarus* | *C. ursinus* | *M. leonina* | *H. grypus* |
| --- | --- | --- | --- | --- | --- | --- | --- | --- |
| *Canis lupus* | 0.1267 | 0.0005 | 0.0070 | 0.0001 | 0.5080 | **0.0000** | 0.0048 | 0.0001 |
| *Vulpes vulpes* | 0.3611 | 0.0160 | 0.2014 | 0.0002 | 0.2447 | **0.0000** | 0.0643 | 0.0008 |
| *Ursus arctos* | 0.0044 | 0.0005 | 0.0010 | 0.0087 | 0.0126 | 0.0003 | **0.0000** | 0.0003 |
| *Ursus maritimus* | 0.0366 | 0.0056 | 0.0078 | 0.0008 | 0.5498 | **0.0000** | 0.0029 | 0.0006 |
| *Melursus ursinus* | 0.0018 | **0.0000** | 0.0002 | 0.0001 | 0.8305 | 0.0009 | 0.0044 | 0.0008 |
| *Ailurus fulgens* | **0.0000** | **0.0000** | 0.0448 | **0.0001** | 0.3132 | 0.0031 | 0.0015 | 0.0002 |
| *Ictonyx striatus* | **0.0000** | **0.0000** | 0.1603 | **0.0000** | 0.4824 | 0.0016 | 0.0002 | 0.0096 |
| *Lutra lutra* | 0.0015 | **0.0000** | 0.5716 | **0.0002** | 0.6001 | 0.0004 | 0.0009 | 0.0026 |
| *Mustela putorius* | **0.0000** | **0.0000** | 0.0408 | 0.0003 | 0.0895 | 0.0563 | 0.0019 | 0.0073 |
| *Meles meles* |  | **0.0000** | 0.0048 | **0.0001** | 0.0967 | 0.0115 | 0.0002 | 0.0221 |
| *Nasua nasua* | 0.60 |  | 0.0123 | **0.0000** | 0.2188 | 0.0019 | **0.0001** | 0.0004 |
| *Potos flavus* | 0.35 | 0.31 |  | 0.0065 | 0.0686 | 0.0053 | 0.0004 | 0.0034 |
| *Procyon lotor* | 0.49 | 0.54 | 0.34 |  | 0.8673 | **0.0001** | 0.0018 | **0.0000** |
| *Odobenus rosmarus* | 0.21 | 0.15 | 0.23 | -0.02 |  | 0.8023 | 0.0129 | 0.1160 |
| *Callorhinus ursinus* | 0.31 | 0.39 | 0.35 | 0.48 | 0.03 |  | 0.0002 | **0.0000** |
| *Mirounga leonina* | 0.46 | 0.48 | 0.44 | 0.39 | 0.31 | 0.46 |  | 0.0002 |
| *Halichoreus grypus* | 0.28 | 0.44 | 0.36 | 0.52 | 0.20 | 0.59 | 0.46 |  |
| *Nandinia binotata* | 0.44 | 0.60 | 0.33 | 0.49 | 0.07 | 0.41 | 0.38 | 0.43 |
| *Felis silvestris* | 0.25 | 0.39 | 0.22 | 0.36 | -0.04 | 0.58 | 0.30 | 0.32 |
| *Panthera tigris* | 0.33 | 0.47 | 0.34 | 0.45 | -0.02 | 0.70 | 0.55 | 0.45 |
| *Genetta tigrina* | 0.33 | 0.48 | 0.29 | 0.53 | -0.09 | 0.60 | 0.52 | 0.51 |
| *Civettictis civetta* | 0.23 | 0.51 | 0.24 | 0.51 | -0.08 | 0.52 | 0.42 | 0.45 |
| *Hyaena hyaena* | 0.27 | 0.48 | 0.33 | 0.51 | 0.10 | 0.60 | 0.48 | 0.47 |
| *Proteles cristatus* | 0.13 | 0.29 | 0.23 | 0.23 | 0.21 | 0.29 | 0.14 | 0.27 |
| *Herpestes ichneumon* | 0.33 | 0.48 | 0.28 | 0.37 | 0.03 | 0.59 | 0.44 | 0.39 |

Table S2 (cont.)

|  | *N. binotata* | *F. silvestris* | *P. tigris* | *G. tigrina* | *C. civetta* | *H. hyaena* | *P. cristatus* |
| --- | --- | --- | --- | --- | --- | --- | --- |
| *Canis lupus* | 0.0083 | **0.0000** | **0.0000** | 0.0001 | **0.0000** | **0.0000** | 0.0426 |
| *Vulpes vulpes* | 0.0709 | **0.0000** | **0.0000** | 0.0015 | 0.0003 | **0.0000** | 0.0221 |
| *Ursus arctos* | 0.0041 | 0.0013 | **0.0001** | 0.0044 | 0.0445 | **0.0000** | 0.0512 |
| *Ursus maritimus* | 0.0292 | **0.0000** | **0.0000** | 0.0037 | 0.0051 | **0.0000** | 0.0527 |
| *Melursus ursinus* | 0.0025 | 0.0018 | 0.0006 | 0.0017 | 0.0007 | 0.0005 | 0.0143 |
| *Ailurus fulgens* | **0.0000** | 0.0063 | 0.0046 | **0.0001** | 0.0002 | 0.0032 | 0.0020 |
| *Ictonyx striatus* | **0.0000** | 0.0031 | **0.0000** | 0.0002 | **0.0000** | 0.0003 | 0.0727 |
| *Lutra lutra* | 0.0020 | 0.0016 | **0.0001** | 0.0004 | 0.0006 | 0.0019 | 0.2501 |
| *Mustela putorius* | 0.0022 | 0.6221 | 0.0738 | 0.0534 | 0.1009 | 0.0983 | 0.8924 |
| *Meles meles* | 0.0004 | 0.0475 | 0.0074 | 0.0084 | 0.0669 | 0.0290 | 0.2824 |
| *Nasua nasua* | **0.0000** | 0.0019 | 0.0001 | **0.0001** | **0.0000** | **0.0001** | 0.0193 |
| *Potos flavus* | 0.0085 | 0.0726 | 0.0070 | 0.0196 | 0.0547 | 0.0082 | 0.0703 |
| *Procyon lotor* | **0.0001** | 0.0037 | 0.0003 | **0.0000** | **0.0000** | **0.0000** | 0.0677 |
| *Odobenus rosmarus* | 0.5869 | 0.7200 | 0.8563 | 0.4929 | 0.5088 | 0.4298 | 0.0945 |
| *Callorhinus ursinus* | 0.0010 | **0.0000** | **0.0000** | **0.0000** | **0.0000** | **0.0000** | 0.0206 |
| *Mirounga leonina* | 0.0024 | 0.0158 | **0.0000** | **0.0000** | 0.0008 | **0.0001** | 0.2735 |
| *Halichoreus grypus* | 0.0005 | 0.0111 | 0.0003 | **0.0000** | 0.0003 | 0.0002 | 0.0277 |
| *Nandinia binotata* |  | 0.0207 | 0.0012 | **0.0000** | **0.0000** | 0.0039 | 0.0136 |
| *Felis silvestris* | 0.29 |  | **0.0000** | 0.0006 | 0.0002 | **0.0000** | 0.0030 |
| *Panthera tigris* | 0.40 | 0.61 |  | **0.0000** | **0.0000** | **0.0000** | 0.0224 |
| *Genetta tigrina* | 0.57 | 0.43 | 0.65 |  | **0.0000** | **0.0000** | 0.0071 |
| *Civettictis civetta* | 0.55 | 0.46 | 0.55 | 0.73 |  | **0.0000** | 0.0005 |
| *Hyaena hyaena* | 0.36 | 0.57 | 0.65 | 0.55 | 0.62 |  | 0.0004 |
| *Proteles cristatus* | 0.31 | 0.37 | 0.28 | 0.33 | 0.43 | 0.44 |  |
| *Herpestes ichneumon* | 0.55 | 0.54 | 0.60 | 0.57 | 0.53 | 0.56 | 0.43 |

Table S3. List of heterochronic shifts in suture closure for each clade and taxon, as determined by Parsimov analysis (consensus of ACCTRAN and DELTRAN).

| **Caniformia** | | | |
| --- | --- | --- | --- |
| Interpremaxillary | L | Intermaxillary, Maxillo-Palatine |  |
| **Canidae** | | | |
| Palato-Alisphenoid | E | Basispheno-Presphenoid, Lacrimo-Frontal, Premaxillo-Nasal, Naso-Frontal |  |
| Palato-Orbitosphenoid | E | Basispheno-Presphenoid, Lacrimo-Frontal, Naso-Frontal |  |
| Premaxillo-Maxillary (f) | E | Maxillo-Palatine, Exoccipito-Squamosal |  |
| Fronto-Parietal | L | Parieto-Squamosal |  |
| ***Canis lupus*** | | | |
| Pterygo-Palatine | E | Maxillo-Palatine, Maxillo-Lacrimal |  |
| ***Vulpes vulpes*** | | | |
| Jugo-Squamosal | E | Lacrimo-Frontal, Premaxillo-Nasal |  |
| Interparietal | L | Basispheno-Basioccipital, Alisphenoid-Orbitosphenoid, Exoccipito-Squamosal |  |
| Parieto-Squamosal | L | Alisphenoid-Orbitosphenoid |  |
| Supraoccipito-Parietal | L | Basispheno-Basioccipital, Alisphenoid-Orbitosphenoid, Exoccipito-Squamosal |  |
| **Ursidae** | | | |
| Interpremaxillary | L | Lacrimo-Frontal,Naso-Frontal |  |
| Basispheno-Presphenoid | L | Internasal, Naso-Frontal |  |
| ***Ursus* (Genus)** | | | |
| Maxillo-Lacrimal | E | Intermaxillary, Maxillo-Palatine, Interpalatine, Basispheno-Basioccipital |  |
| Premaxillo-Nasal | E | Basispheno-Presphenoid, Palato-Orbitosphenoid |  |
| Naso-Frontal | E | Lacrimo-Frontal, Internasal |  |
| Exoccipito-Squamosal | E | Basispheno-Basioccipital |  |
| ***Ursus arctos*** | | | |
| Premaxillo-Maxillary (v) | E | Basispheno-Basioccipital, Fronto-Parietal, Fronto-Squamosal |  |
| Maxillo-Palatine | E | Intermaxillary, Basispheno-Basioccipital, Fronto-Parietal, Fronto-Squamosal |  |
| Pterygo-Palatine | E | Basispheno-Basioccipital, Orbitospheno-Frontal, Interfrontal, Fronto-Parietal, Fronto-Squamosal |  |
| Alispheno-Squamosal | L | Intermaxillary, Interpalatine, Palato-Alisphenoid, Orbitospheno-Frontal, Palato-Orbital, Maxillo-Jugal, Premaxillo-Nasal |  |
| Alisphenoid-Orbitosphenoid | L | Intermaxillary, Interpalatine, Orbitospheno-Frontal |  |
| Alisphenoid-Orbitosphenoid | L | Intermaxillary, Interpalatine, Orbitospheno-Frontal |  |
| Interfrontal | E | Premaxillo-Maxillary (v), Intermaxillary, Basispheno-Basioccipital, Fronto-Parietal, Fronto-Squamosal |  |
| Parieto-Squamosal | L | Intermaxillary, Interpalatine, Orbitospheno-Frontal |  |
| ***Ursus maritimus*** | | | |
| Interfrontal | L | Maxillo-Palatine, Pterygo-Palatine |  |
| ***Melursus ursinus*** | | | |
| Mandibular Symphysis | E | Interpremaxillary, Maxillo-Palatine, Palato-Alisphenoid, Basispheno-Basioccipital, Basispheno-Presphenoid, Alisphenoid-Orbitosphenoid, Palato-Orbitosphenoid, Lacrimo-Frontal, Premaxillo-Nasal, Internasalal, Interfrontal |  |
| Intermaxillary | E | Maxillo-Palatine, Basispheno-Basioccipital, Alisphenoid-Orbitosphenoid, Fronto-Parietal |  |
| Interpalatine | E | Maxillo-Palatine, Basispheno-Basioccipital, Alisphenoid-Orbitosphenoid, Fronto-Parietal |  |
| Pterygo-Palatine | L | Basispheno-Presphenoid, Lacrimo-Frontal, Naso-Frontal |  |
| Alispheno-Squamosal | E | Basispheno-Basioccipital, Fronto-Parietal, Interparietal, Parieto-Squamosal, Supraoccipito-Parietal |  |
| Jugo-Squamosal | E | Interpremaxillary, Premaxillo-Nasal |  |
| Fronto-Squamosal | E | Basispheno-Basioccipital, Fronto-Parietal, Interparietal |  |
| Parieto-Squamosal | E | Basispheno-Basioccipital, Fronto-Parietal |  |
| **Mustelidae + Pinnipedia** | | | |
| Pterygo-Palatine | E | Premaxillo-Maxillary (v), Maxillo-Palatine |  |
| Interparietal | L | Fronto-Parietal, Fronto-Squamosal, Parieto-Squamosal |  |
| Exoccipito-Squamosal | L | Basispheno-Presphenoid |  |
| **Musteloidea** | | | |
| Basispheno-Basioccipital | L | Alispheno-Squamosal, Orbitospheno-Frontal, Maxillo-Lacrimal, Interfrontal, Fronto-Squamosal |  |
| Supraoccipito-Parietal | L | Premaxillo-Maxillary (v), Alispheno-Squamosal, Interfrontal, Fronto-Parietal, Interpari, Fronto-Squamosal |  |
| Supraoccipito-Squamosal | L | Intermaxillary, Alispheno-Squamosal, Alisphenoid-Orbitosphenoid, Orbitospheno-Frontal, Premaxillo-Maxillary (f), Interfrontal |  |
| Exoccipito-Squamosal | L | Palato-Alisphenoid, Maxillo-Jugal |  |
| ***Ailurus fulgens*** | | | |
| Interpremaxillary | E | Alisphenoid-Orbitosphenoid, Interfrontal |  |
| Maxillo-Palatine | E | Premaxillo-Maxillary (v), Interfrontal, Interparietal |  |
| Pterygo-Palatine | E | Interfrontal, Parieto-Squamosal, Exoccipital-Supraoccipital, Exoccipito-Basioccipitalcc |  |
| Lacrimo-Frontal | L | Palato-Alisphenoid |  |
| **Mustelidae + Procyonidae** | | | |
| Premaxillo-Maxillary (f) | E | Supraoccipito-Parietal |  |
| Internasal | L | Premaxillo-Nasal |  |
| Interfrontal | E | Parieto-Squamosal |  |
| **Mustelidae** | | | |
| Interpremaxillary | L | Basispheno-Presphenoid, Lacrimo-Frontal |  |
| Premaxillo-Maxillary (v) | L | Fronto-Parietal |  |
| Interpalatine | E | Intermaxillary |  |
| **Mustelidae without *Meles meles*** | | | |
| Premaxillo-Maxillary (v) | L | Supraoccipito-Parietal |  |
| Premaxillo-Maxillary (f) | L | Premaxillo-Maxillary (v), Supraoccipito-Parietal |  |
| ***Ictonyx striatus*** | | | |
| Palato-Alisphenoid | E | Orbitospheno-Frontal, Maxillo-Lacrimal |  |
| Interfrontal | L | Parieto-Squamosal |  |
| Fronto-Parietal | L | Alispheno-Squamosal, Alisphenoid-Orbitosphenoid, Fronto-Squamosal |  |
| Parieto-Squamosal | L | Alispheno-Squamosal, Alisphenoid-Orbitosphenoid, Interparietal |  |
| ***Lutra lutra + Mustela putorius*** | | | |
| Alisphenoid-Orbitosphenoid | E | Pterygo-Palatine, Alispheno-Squamosal |  |
| Orbitospheno-Frontal | E | Pterygo-Palatine, Alispheno-Squamosal, Alisphenoid-Orbitosphenoid |  |
| Lacrimo-Frontal | E | Supraoccipito-Parietal |  |
| ***Lutra lutra*** | | | |
| Premaxillo-Maxillary (v) | L | Maxillo-Lacrimal, Supraoccipito-Parietal |  |
| Interpalatine | E | Pterygo-Palatine, Alispheno-Squamosal, Alisphenoid-Orbitosphenoid, Orbitospheno-Frontal |  |
| Palato-Alisphenoid | E | Maxillo-Lacrimal, Lacrimo-Frontal, Supraoccipito-Parietal |  |
| Basispheno-Presphenoid | E | Palato-Alisphenoid, Basispheno-Basioccipital, Maxillo-Lacrimal, Lacrimo-Frontal, Supraoccipito-Parietal |  |
| Palato-Orbitosphenoid | E | Interpremaxillary, Jugo-Squamosal, Premaxillo-Nasal |  |
| Maxillo-Jugal | L | Premaxillo-Maxillary (v), Intermaxillary, Maxillo-Lacrimal, Lacrimo-Frontal, Supraoccipito-Parietal |  |
| Premaxillo-Maxillary (f) | L | Maxillo-Lacrimal, Lacrimo-Frontal, Supraoccipito-Parietal |  |
| Interfrontal | L | Maxillo-Lacrimal, Lacrimo-Frontal, Supraoccipito-Parietal |  |
| Fronto-Parietal | L | Pterygo-Palatine, Alispheno-Squamosal, Alisphenoid-Orbitosphenoid |  |
| Supraoccipito-Squamosal | E | Basispheno-Basioccipital, Jugo-Squamosal, Premaxillo-Nasal |  |
| Exoccipito-Squamosal | E | Interpremaxillary, Palato-Alisphenoid, Basispheno-Basioccipital, Basispheno-Presphenoid, Maxillo-Lacrimal, Jugo-Squamosal, Lacrimo-Frontal, Premaxillo-Nasal, Internasal, Supraoccipito-Parietal, Supraoccipito-Squamosal |  |
| Supraoccipito-Squamosal | E | Basispheno-Basioccipital, Jugo-Squamosal, Premaxillo-Nasal |  |
| Exoccipito-Squamosal | E | Interpremaxillary, Palato-Alisphenoid, Basispheno-Basioccipital, Basispheno-Presphenoid, Maxillo-Lacrimal, Jugo-Squamosal, Lacrimo-Frontal, Premaxillo-Nasal, Internasal, Supraoccipito-Parietal, Supraoccipito-Squamosal |  |
| Exoccipital-Supraoccipital | L | Pterygo-Palatine, Alispheno-Squamosal, Alisphenoid-Orbitosphenoid, Exoccipito-Basioccipital |  |
| ***Mustela putorius*** | | | |
| Intermax | E | Maxillo-Lacrimal, Lacrimo-Frontal, Supraoccipito-Parietal |  |
| Naso-Frontal | E | Premaxillo-Maxillary (v), Intermaxillary, Maxillo-Lacrimal, Lacrimo-Frontal, Premaxillo-Maxillary (f), Supraoccipito-Parietal |  |
| Parieto-Squamosal | L | Pterygo-Palatine, Alispheno-Squamosal, Alisphenoid-Orbitosphenoid, Orbitospheno-Frontal |  |
| ***Meles meles*** | | | |
| Interpremaxillary | L | Internasal, Exoccipito-Squamosal |  |
| Alisphenoid-Orbitosphenoid | L | Orbitospheno-Frontal, Maxillo-Lacrimal, Lacrimo-Frontal |  |
| ***Nasua nasua + Procyon lotor*** | | | |
| Alispheno-Squamosal | E | Fronto-Parietal |  |
| ***Nasua nasua*** | | | |
| Interpalatine | E | Intermaxillary, Supraoccipito-Parietal |  |
| Pterygo-Palatine | E | Alispheno-Squamosal, Interfrontal, Fronto-Parietal, Supraoccipito-Parietal |  |
| Premaxillo-Maxillary (f) | L | Premaxillo-Maxillary (v), Maxillo-Palatine |  |
| ***Procyon lotor*** | | | |
| Interpremaxillary | E | Maxillo-Jugal, Supraoccipito-Squamosal |  |
| Pterygo-Palatine | L | Intermaxillary, Lacrimo-Frontal |  |
| Basispheno-Basioccipital | E | Maxillo-Palatine, Fronto-Parietal |  |
| Basispheno-Presphenoid | E | Palato-Alisphenoid, Lacrimo-Frontal |  |
| Alisphenoid-Orbitosphenoid | E | Maxillo-Palatine, Alispheno-Squamosal, Fronto-Parietal |  |
| Orbitospheno-Frontal | E | Maxillo-Palatine, Alisphenoid-Orbitosphenoid, Premaxillo-Maxillary (f), Fronto-Parietal |  |
| Palato-Orbitosphenoid | L | Jugo-Squamosal, Premaxillo-Nasal |  |
| Premaxillo-Maxillary (f) | E | Alispheno-Squamosal, Alisphenoid-Orbitosphenoid |  |
| Fronto-Squamosal | L | Premaxillo-Maxillary (v), Alispheno-Squamosal, Interparietal |  |
| Supraoccipito-Parietal | L | Intermaxillary, Maxillo-Jugal |  |
| ***Potos flavus*** | | | |
| MandSymph | E | Interpremaxillary, Premaxillo-Maxillary (v), Palato-Orbitosphenoid, Jugo-Squamosal, Maxillo-Jugal, Premaxillo-Maxillary (f), Premaxillo-Nasal, Internasal, Naso-Frontal, Supraoccipito-Parietal, Supraoccipito-Squamosal, Exoccipito-Squamosal |  |
| Pterygo-Palatine | L | Alisphenoid-Orbitosphenoid |  |
| Alispheno-Squamosal | L | Premaxillo-Maxillary (v), Interfrontal |  |
| Fronto-Squamosal | L | Premaxillo-Maxillary (v), Interfrontal, Interparietal |  |
| ***Odobenus rosmarus + Callorhinus ursinus*** | | | |
| Premaxillo-Maxillary (v) | L | Interfrontal, Fronto-Parietal |  |
| Maxillo-Jugal | E | Maxillo-Palatine, Alisphenoid-Orbitosphenoid, Orbitospheno-Frontal |  |
| Supraoccipito-Squamosal | E | Fronto-Squamosal, Parieto-Squamosal |  |
| Exoccipito-Squamosal | E | Alispheno-Squamosal, Alisphenoid-Orbitosphenoid |  |
| ***Odobenus rosmarus*** | | | |
| MandSymph | E | Intermaxillary, Maxillo-Palatine, Interpalatine, Palato-Alisphenoid, Basispheno-Presphenoid |  |
| Interpremaxillary | L | Intermaxillary, Basispheno-Presphenoid, Maxillo-Jugal |  |
| Basispheno-Basioccipital | L | Pterygo-Palatine, Maxillo-Jugal, Interfrontal |  |
| Alispheno-Squamosal | L | Interpremaxillary, Premaxillo-Maxillary (v), Intermaxillary, Maxillo-Palatine, Interpalatine, Pterygo-Palatine, Palato-Alisphenoid, Basispheno-Presphenoid, Maxillo-Jugal |  |
| Alisphenoid-Orbitosphenoid | L | Intermaxillary, Maxillo-Palatine, Interpalatine, Palato-Alisphenoid, Basispheno-Presphenoid |  |
| Orbitospheno-Frontal | L | Intermaxillary, Maxillo-Palatine, Interpalatine, Palato-Alisphenoid, Basispheno-Presphenoid |  |
| Premaxillo-Maxillary (f) | E | Interfrontal |  |
| Premaxillo-Nasal | E | Intermaxillary, Maxillo-Palatine, Interpalatine, Pterygo-Palatine, Palato-Alisphenoid, Basispheno-Presphenoid, Maxillo-Lacrimal, Lacrimo-Frontal, Maxillo-Jugal, Premaxillo-Maxillary (f), Naso-Frontal, Interfrontal |  |
| Internasal | E | Intermaxillary, Maxillo-Palatine, Basispheno-Presphenoid, Lacrimo-Frontal, Maxillo-Jugal |  |
| Naso-Frontal | E | Intermaxillary, Maxillo-Palatine, Interpalatine, Basispheno-Presphenoid, Maxillo-Jugal |  |
| Fronto-Parietal | L | Pterygo-Palatine, Interfrontal |  |
| Interparietal | L | Intermaxillary, Basispheno-Presphenoid, Maxillo-Jugal, Interfrontal |  |
| Fronto-Squamosal | L | Interpremaxillary, Intermaxillary, Maxillo-Palatine, Interpalatine, Pterygo-Palatine, Basispheno-Presphenoid, Maxillo-Lacrimal, Lacrimo-Frontal, Maxillo-Jugal, Interfrontal, Interparietal |  |
| Parieto-Squamosal | L | Interpremaxillary, Intermaxillary, Maxillo-Palatine, Interpalatine, Pterygo-Palatine,Palato-Alisphenoid, Basispheno-Presphenoid, Maxillo-Jugal, Interfrontal, Interparietal |  |
| Supraoccipito-Squamosal | L | Intermaxillary, Maxillo-Palatine, Interpalatine, Pterygo-Palatine, Basispheno-Presphenoid, Maxillo-Jugal, Interfrontal |  |
| Exoccipito-Squamosal | L | Maxillo-Palatine, Interpalatine, Palato-Alisphenoid, Maxillo-Jugal |  |
| ***Callorhinus ursinus*** | | | |
| Premaxillo-Maxillary (v) | L | Interpremaxillary, Orbitospheno-Frontal, Interparietal |  |
| Interpalatine | E | Intermaxillary, Maxillo-Palatine, Basispheno-Presphenoid |  |
| Palato-Alisphenoid | E | Intermaxillary, Maxillo-Palatine, Naso-Frontal |  |
| Basispheno-Basioccipital | E | Supraoccipito-Parietal, Exoccipito-Basioccipitalcc |  |
| Palato-Orbitosphenoid | E | Intermaxillary, Interpalatine |  |
| Maxillo-Lacrimal | L | Pterygo-Palatine, Basispheno-Presphenoid, Lacrimo-Frontal, Premaxillo-Maxillary (f), Interfrontal |  |
| Lacrimo-Frontal | L | Intermaxillary, Basispheno-Presphenoid |  |
| Exoccipito-Squamosal | E | Interpremaxillary, Intermaxillary, Pterygo-Palatine, Basispheno-Presphenoid |  |
| ***Halichoerus grypus*** | | | |
| Intermax | E | Premaxillo-Maxillary (f) |  |
| Palato-Orbitosphenoid | E | Exoccipito-Squamosal |  |
| Lacrimo-Frontal | E | Premaxillo-Maxillary (f) |  |
| **Feliformia** | | | |
| Pterygo-Palatine | E | Premaxillo-Maxillary (v), Fronto-Squamosal, Parieto-Squamosal, Supraoccipito-Squamosal |  |
| Basispheno-Presphenoid | L | Palato-Alisphenoid, Palato-Orbitosphenoid |  |
| Naso-Frontal | E | Lacrimo-Frontal |  |
| Fronto-Parietal | L | Alispheno-Squamosal, Interfrontal |  |
| Supraoccipito-Parietal | L | Interparietal |  |
| ***Nandinia binotata*** | | | |
| Premaxillo-Maxillary (v) | E | Alisphenoid-Orbitosphenoid, Interfrontal |  |
| Intermax | E | Alispheno-Squamosal, Fronto-Parietal |  |
| Basispheno-Basioccipital | L | Orbitospheno-Frontal, Maxillo-Lacrimal, Internasal, Supraoccipito-Squamosal |  |
| Supraoccipito-Parietal | L | Orbitospheno-Frontal, Interfrontal, Fronto-Parietal, Fronto-Squamosal, Exoccipital-Squamosal |  |
| **Feliformia without *Nandinia binotata*** | | | |
| Palato-Orbitosphenoid | E | Maxillo-Lacrimal, Premaxillo-Nasal, Internasal, Naso-Frontal |  |
| Premaxillo-Maxillary (f) | E | Maxillo-Lacrimal, Internasal |  |
| **Felidae** | | | |
| Interpremaxillary | L | Maxillo-Palatine, Interpalatine, Palato-Alisphenoid, Maxillo-Jugal, Premaxillo-Maxillary (f) |  |
| Premaxillo-Maxillary (v) | L | Maxillo-Palatine, Maxillo-Jugal |  |
| Intermax | L | Maxillo-Palatine, Interpalatine, Maxillo-Jugal, Premaxillo-Maxillary (f) |  |
| ***Felis silvestris*** | | | |
| Basispheno-Presphenoid | E | Palato-Orbitosphenoid, Premaxillo-Maxillary (f) |  |
| Interfrontal | L | Premaxillo-Maxillary (v), Fronto-Parietal |  |
| Interparietal | L | Premaxillo-Maxillary (v), Basispheno-Basioccipital, Alisphenoid-Orbitosphenoid, Orbitospheno-Frontal, Fronto-Parietal, Fronto-Squamosal, Parieto-Squamosal, Supraoccipito-Parietal, Supraoccipito-Squamosal |  |
|  | | | |
| ***Panthera tigris*** | | | |
| Intermax | L | MandSymph, Lacrimo-Frontal, Premaxillo-Nasal |  |
| Orbitospheno-Frontal | E | Alisphenoid-Orbitosphenoid, Fronto-Parietal |  |
| **Viverridae** | | | |
| Palato-Orbitosphenoid | E | Maxillo-Palatine, Interpalatine, Maxillo-Jugal |  |
| Supraoccipito-Parietal | L | Premaxillo-Maxillary (v), Alispheno-Squamosal, Alisphenoid-Orbitosphenoid, Orbitospheno-Frontal, Fronto-Parietal, Exoccipito-Squamosal |  |
| Supraoccipito-Squamosal | L | Premaxillo-Maxillary (v), Alispheno-Squamosal, Alisphenoid-Orbitosphenoid, Orbitospheno-Frontal |  |
| Exoccipito-Squamosal | L | Premaxillo-Maxillary (v) |  |
| ***Genetta tigrina*** | | | |
| Alispheno-Squamosal | L | Fronto-Parietal |  |
| Orbitospheno-Frontal | E | Premaxillo-Maxillary (v), Basispheno-Basioccipital, Fronto-Parietal, Fronto-Squamosal |  |
| Palato-Orbitosphenoid | E | Fronto-Parietal, Supraoccipito-Parietal, Exoccipito-Squamosal |  |
| Internasal | E | Basispheno-Presphenoid, Maxillo-Lacrimal |  |
| ***Civettictis civetta*** | | | |
| Interpalatine | L | Maxillo-Lacrimal, Internasal |  |
| Palato-Alisphenoid | E | Interpremaxillary, Supraoccipito-Parietal, Exoccipito-Squamosal |  |
| Basispheno-Basioccipital | L | Premaxillo-Maxillary (v), Alisphenoid-Orbitosphenoid |  |
| Interfrontal | L | Premaxillo-Maxillary (v), Fronto-Parietal |  |
| Parieto-Squamosal | L | Alisphenoid-Orbitosphenoid, Fronto-Squamosal |  |
| Supraoccipito-Squamosal | L | Maxillo-Palatine, Supraoccipito-Parietal, Exoccipito-Squamosal |  |
| **Hyaenidae** | | | |
| Palato-Alisphenoid | E | Fronto-Squamosal, Parieto-Squamosal, Supraoccipito-Parietal, Supraoccipito-Squamosal |  |
| Basispheno-Basioccipital | E | Parieto-Squamosal |  |
| Basispheno-Presphenoid | E | Maxillo-Palatine, Orbitospheno-Frontal, Fronto-Parietal |  |
| Premaxillo-Maxillary (f) | E | Maxillo-Palatine, Alispheno-Squamosal, Orbitospheno-Frontal, Fronto-Parietal, Fronto-Squamosal |  |
| ***Hyaena hyaena*** | | | |
| Interpremaxillary | L | Alisphenoid-Orbitosphenoid, Fronto-Parietal, Exoccipito-Squamosal |  |
| Intermax | L | Maxillo-Palatine |  |
| Pterygo-Palatine | L | Basispheno-Basioccipital, Interparietal |  |
| ***Proteles cristatus*** | | | |
| MandSymph | E | Interpremaxillary, Intermaxillary, Maxillo-Palatine, Interpalatine, Basispheno-Presphenoid, Alisphenoid-Orbitosphenoid, Palato-Orbitosphenoid, Maxillo-Lacrimal, Jugo-Squamosal, Maxillo-Jugal, Premaxillo-Maxillary (f), Premaxillo-Nasal, Internasal, Parieto-Squamosal, Supraoccipito-Squamosal |  |
| Interpremaxillary | E | Parieto-Squamosal, Supraoccipito-Squamosal |  |
| Premaxillo-Maxillary (v) | E | Alisphenoid-Orbitosphenoid, Parieto-Squamosal, Supraoccipito-Squamosal |  |
| Interpalatine | E | Maxillo-Palatine, Supraoccipito-Squamosal |  |
| Basispheno-Presphenoid | E | Alisphenoid-Orbitosphenoid, Premaxillo-Maxillary (f), Supraoccipito-Squamosal |  |
| Maxillo-Lacrimal | E | Interpremaxillary, Intermaxillary, Maxillo-Palatine, Basispheno-Presphenoid, Alispheno-Orbitosphenoid, Premaxillo-Maxillary (f), Parieto-Squamosal, Supraoccipito-Squamosal |  |
| Maxillo-Jugal | E | Maxillo-Palatine, Alisphenoid-Orbitosphenoid, Supraoccipito-Squamosal |  |
| Supraoccipito-Parietal | L | Alispheno-Squamosal, Premaxillo-Maxillary (f), Fronto-Squamosal |  |
| ***Herpestes ichneumon*** | | | |
| Intermax | E | Interpremaxillary, Palato-Alisphenoid, Interparietal |  |
| Lacrimo-Frontal | E | MandSymph, Premaxillo-Nasal, Naso-Frontal |  |
